# Effect of Combined Infrared Hot Air Drying on Yam Slices: Drying Kinetics, Energy Consumption, Microstructure, and Nutrient Composition

**DOI:** 10.3390/foods12163048

**Published:** 2023-08-14

**Authors:** Jikai Zhang, Xia Zheng, Hongwei Xiao, Yican Li, Taoqing Yang

**Affiliations:** 1 College of Mechanical and Electrical Engineering, Shihezi University, Shihezi 832003, China; 15154159806@163.com (J.Z.); 20202109079@stu.shzu.edu.cn (Y.L.); 20212009002@stu.shzu.edu.cn (T.Y.); 2 Key Laboratory of Northwest Agricultural Equipment, Ministry of Agriculture and Rural Affairs, Shihezi 832003, China; 3 Key Laboratory of Modern Agricultural Machinery Corps, Shihezi 832003, China; 4 College of Engineering, China Agricultural University, Beijing 100080, China; xhwcaugxy@163.com

**Keywords:** yam slices, infrared hot air combined drying, hot air drying, drying kinetics, quality

## Abstract

Using hot air drying (HAD) and combined infrared hot air drying (IR-HAD) test devices, the drying kinetics, unit energy consumption, color difference values, rehydration rate, microstructure, and changes in polysaccharide and allantoin contents of yam slices were examined at various temperatures (50 °C, 55 °C, 60 °C, 65 °C, and 70 °C). The findings demonstrated that each of the aforementioned parameters was significantly impacted by the drying temperature. IR-HAD dries quicker and takes less time to dry than HAD. The *D_e__ff_* of IR-HAD is higher than that of HAD at the same temperature and increases with the increase in temperature. The activation energy required for IR-HAD (26.35 kJ/mol) is lower than that required for HAD (32.53 kJ/mol). HAD uses more energy per unit than IR-HAD by a factor of greater than 1.3. Yam slices treated with IR-HAD had higher microscopic porosity, better rehydration, lower color difference values, and higher polysaccharide and allantoin levels than HAD-treated yam slices. The IR-HAD at 60 °C had the greatest comprehensive rating after a thorough analysis of the dried yam slices using the coefficient of variation method. Three statistical indicators were used to evaluate six thin-layer drying models, and the Weibull model was most applicable to describe the variation of drying characteristics of yam slices.

## 1. Introduction

Yam, a twining vine that is rich in polysaccharides, allantoin, protein, and other nutrients, is useful for treating asthma and autoimmune diseases as well as alleviating diarrhea [1]. The majority of the world’s yam species, which number over 600, are found in Africa, Asia, and the Americas. Among these, 93 species, or nearly one-sixth of all species worldwide, are found in China [2]. However, fresh yams with great crispness and up to 84% moisture content are very prone to mechanical damage, fermentation, and mold during sale, processing, transit, and storage, which results in wasted food and environmental pollution [3]. Yam shelf life is increased and mold damage is avoided by drying them [4]. However, yams undergo irreversible biological and chemical processes, as well as structural and physicochemical alterations, after drying [5]. It is possible to minimize the loss of nutrients from yam by selecting an appropriate drying technique.

Scholars from both home and abroad have investigated the drying of yam. Hot air drying (HAD) is one of the most extensively used drying technologies due to its ease of use, low cost, broad applicability, and easy control [6]. However, during the drying process, the material is typically subjected to high temperatures and oxygen environments, which can cause surface hardening crust, browning, and other phenomena, and when the drying rate is poor, the product quality varies. Although the drying quality of vacuum freezing is good, the equipment is expensive and unsuitable for industrial production [7]. Microwave drying causes loosening of the material tissue structure, which can result in significant nutritional loss [8]. Infrared drying is more advantageous in terms of product quality and drying pace, but it also consumes more energy [9]. Infrared drying has more advantages in terms of product quality and drying rate [9]. Considering the low cost of hot air drying (HAD), the simplicity of operation, and the possibility of mass production, combined with the advantages of high radiation efficiency, fast heating rate, good quality, and selectivity between the radiating body and the heated material, infrared heating and drying technology are used. Infrared and hot air drying can be coupled and complement one another [3].

IR-HAD of fruits and vegetables, such as carrots [10], sweet potato [11], mango slices [12], and ginseng root slices [13], is gaining popularity among researchers. Xu et al. [14] dried chrysanthemums using a combination of infrared hot air drying (IR-HAD) and hot air drying (HAD) techniques and discovered that IR-HAD was more effective in terms of reducing energy consumption and improving shrinkage, rehydration capacity, and cell microstructure and that the Page model fit the dehydration process better. Zhang et al. [15] investigated the effects of combined short- and medium-wave infrared gas jet impingement drying on the drying properties and quality of loofah slices with various settings. Temperature and slice thickness were discovered to be the most significant factors influencing drying time, and combined short- and medium-wave infrared gas jet impingement drying had significant advantages in reducing drying time and quality (color and total saponin) when compared to HAD under the same conditions. Xu et al. [5] investigated the influence of several temperatures on the drying characteristics and quality of goldenrod and determined that far-infrared mixed with hot air drying reduced drying time while improving color, scent, and active components. At present, there is a lack of relevant research reports on yam IR-HAD, and the above materials show that this combined drying technology has good application prospects.

The pre-test results show that drying temperature is the most important element influencing the drying characteristics and quality of yam slices. As a result, the following were the study’s objectives:

1. To investigate the effects of IR-HAD and HAD at various temperatures (50 °C, 55 °C, 60 °C, 65 °C, and 70 °C) on the drying process, unit energy consumption, color, rehydration rate, microstructure, and polysaccharide and allantoin content of yam slices.
2. To calculate the effective moisture diffusion coefficient and drying activation energy during the drying process of yam slices and establish the corresponding mathematical models and verify them through experiments.

## 2. Materials and Methods

### 2.1. Materials

Fresh desert yams were obtained from Shihezi wholesale farmers’ market. Yams from the same batch that were free of diseases and insects, mechanical damage, mold, and rot were chosen and stored in a refrigerator at 4 ± 1 °C. Prior to the test, mud and grime were washed off the yams’ surfaces with tap water, then the yams were peeled manually and sliced into uniform slices of 6 mm thickness with a slicer. To remove incorrectly proportioned yam pieces, vernier calipers were used to measure (precision of 0.02 mm) each sample numerous times. By baking the samples for 24 h in a hot air oven at 105 °C, the average initial moisture content was determined to be 84.28 ± 1.32% [16].

### 2.2. Test Method

**Figure 1:**
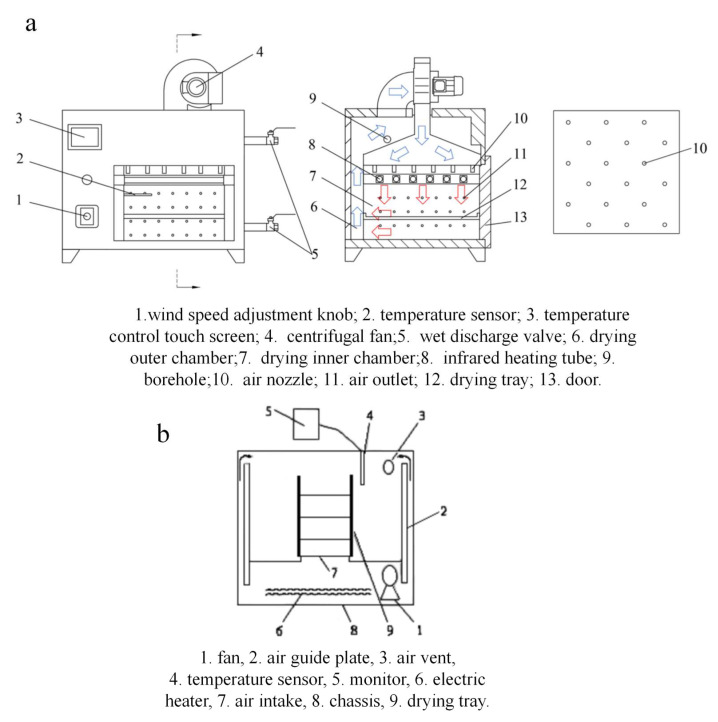
Structure diagrams of an infrared hot air drier (**a**) and a hot air dryer (**b**). Note: The arrows in Figure 1 indicate the circulation of hot air in the dryer, the red arrow is the inflow and the blue arrow is the outflow.

**Figure 2:**
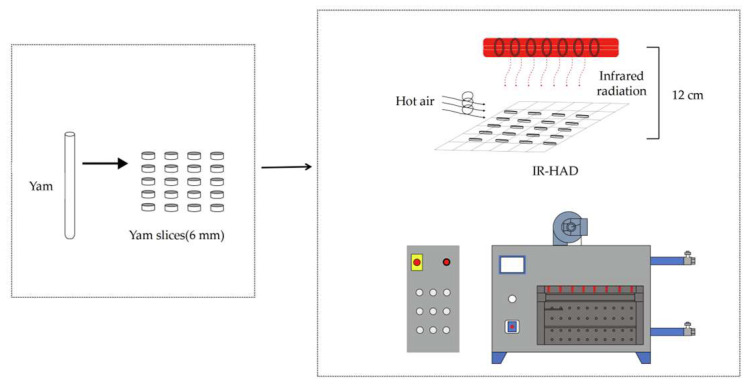
Depicts the drying of yam slices.

**Figure 3:**
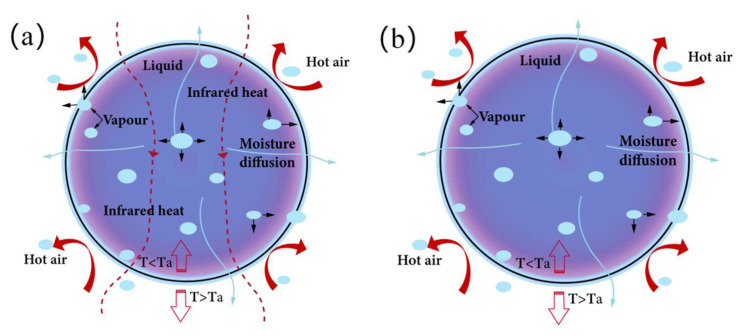
Water evaporation mechanism of yam slices using IR-HAD (**a**) and HAD (**b**). Note: Arrows indicate hot air heat transfer.

In order to realize the best drying effect of yam slices, an infrared hot air combined dryer (STC, Taizhou Shengtaike Infrared Technology Co., Ltd., Taizhou, China; temperature accuracy of ±0.1 °C, power range of 0~2 kW) and an electrothermal constant-temperature air blower drying oven (DHG-9070A, temperature control range (10~250 °C), temperature control accuracy of ±0.1 °C, Shanghai Yiheng Science & Technology Co., Ltd., Shanghai, China) were adjusted to the desired temperatures (50 °C, 55 °C, 60 °C, 65 °C, and 70 °C) (Figure 1). Using an anemometer (TES-1340,Taiwan Tai-Style Electronics Industry Co., Taiwan, China), the wind speed was measured at the nozzle and set to 3 ± 0.14 m/s. Figure 2 shows yam slices (150 g) with a slicing thickness of 6 mm evenly dispersed in a single layer on a 37 cm × 20 cm tray and dried in a drier. The weight loss of the samples was measured every 15 min with an electronic balance (BSM-4200.2,Shanghai Zhuojing Electronic Technology Co., Shanghai, China, sensitivity 0.01 g) until the target moisture content (0.13 kg/kg) was reached. The samples were immediately chilled and vacuum-sealed in low-density polyethylene (LDPE) bags for subsequent examination, and each set of tests was repeated three times to verify accuracy. The method of IR-HAD and HAD moisture evaporation from yam slices is depicted in Figure 3.

### 2.3. Drying Characteristics

The moisture ratio (*MR*) and drying rate (*DR*) of yam slices were associated with the water loss of yam slices during the drying process, and the *MR-t* and *DR-M_t_* curves were measured every 15 min.

The formula for calculating the moisture ratio [17] is shown in Equation (1).

(1) $$MR=\frac{M_{t}}{M_{0}}MR=\frac{M_{t}}{M_{0}}$$

where *MR* denotes the moisture ratio, *M_t_* denotes the moisture content at time *t* in kg/kg d.b., and *M*_0_ denotes the initial moisture content in kg/kg d.b.

Equation (2) is used to calculate the drying rate of yam slices [18].

(2) $$DR=\frac{M_{t1}-M_{t2}}{t_{1}-t_{2}}$$

where *M_t_*_1_ denotes the moisture content of dry basis at *t*_1_ in g/g and *M_t_*_1_ denotes the moisture content of dry basis at *t*_1_ in g/g.

### 2.4. Moisture Diffusion Coefficient Effective

For temperature, which is the main factor affecting the effective water diffusion coefficient, the law can be described by Fick’s second law [19], which is calculated as Equation (3).

(3) $$MR=\frac{M_{t}}{M_{0}}\approx \frac{8}{\pi^{2}}exp\left(\frac{D_{eff}\pi^{2}}{L^{2}}t\right)$$

The previous Equation (3) is reduced further by taking the natural logarithm of both sides, as shown in Equation (4).

(4) $$lnMR=ln\frac{8}{\pi^{2}}-\frac{{D_{eff}\pi }^{2}}{L^{2}}t$$

According to Equation (4), the logarithmic value of the moisture ratio is linearly proportional to the drying time *t*. Equation (5) depicts the link between the slope of this line k and the effective moisture diffusion coefficient *D_e__ff_*.

(5) $$D_{eff}=-\frac{L^{2}}{\pi^{2}}k$$

where *D_e__ff_* is the effective moisture diffusion coefficient, m^2^/s; k is the slope of the equation; and *L* is the thickness of yam slices, mm.

### 2.5. Drying Activation Energy

The activation energy reflects the ease with which material can be dewatered. The link between the temperature of yam slices and the effective moisture diffusion coefficient, which is derived as Equation (6), can be expressed using the Arrhenius equation [20].

(6) $$D_{eff}=lnD_{0}-\frac{Ea}{R\left(T+273.15\right)}$$

where *D_eff_* is the effective moisture diffusion coefficient, m^2^/s; *D*_0_ is the diffusion base, m^2^/s; *Ea* is the drying activation energy, J/mol; *R* is the molar constant of gas, 8.314 J/mol·K; and *T* is the drying temperature, °C.

### 2.6. Unit Energy Consumption

The energy required to remove a unit mass of water is calculated according to Equation (7) [21].

(7) $$\varphi =\frac{Q}{M}$$

where *φ* is the unit energy consumption, kJ·h/kg; *Q* is the total energy consumption measured by the intelligent meter at the end of drying, kJ·h; and *M* is the dehydrated mass of yam slices at the end of drying, kg.

### 2.7. Determination of Color and Luster

The dried yam slices were taken, and their colorimetric values (L*, a*, b*) were measured according to the CIELAB colorimeter system using a SMY-2000SF colorimeter (Beijing Sheng Ming Yang Technology Development Co., Ltd., Beijing, China). The color difference value Δ*E* between the dried treatment group and the fresh sample was calculated according to the following equation [22].

(8) $$∆E=\sqrt{{\left(L-L^{*}\right)}^{2}+{\left(a-a^{*}\right)}^{2}+{\left(b-b^{*}\right)}^{2}}$$

where *L*, *a*, and *b* denote the brightness, red-green value, and yellow-blue value of fresh yam; L*, a*, and b* denote the brightness, red-green value, and yellow-blue value of dried yam slices.

### 2.8. Determination of Rehydration Ratio

A beaker containing distilled water was placed in a 40 °C water bath. When the temperature of the distilled water had stabilized, 5 g of yam slices were added to 50 mL of the liquid. They were removed after soaking for 2 h, and the surface was dried with absorbent paper before being weighed using an analytical scale. The compound water ratio [23] was calculated as follows:

(9) $$R_{r}=\frac{m_{1}}{m_{2}}$$

where *R_r_* is the rehydration ratio, g/g; *m*_1_ is the mass of the sample after rehydration, g; *m*_2_ is the mass of the sample before rehydration, g.

### 2.9. Microstructure

A typical slice was chosen from three sets of yam slice samples and cut off immediately after quick-freezing with liquid nitrogen to form a crisp cross-section naturally. The samples were glued to the sample trays with carbon conductive adhesive and sprayed with gold before being scanned by electron microscopy (Scanning Electronic Microscopy, SEM) [24], and representative images were selected for preservation and further analysis.

### 2.10. Polysaccharide Composition

The polysaccharide content was determined using the method described by Zhou et al. [25]. A total of 3 g of yam sample was weighed accurately, crushed, ground into powder, run through a 60-mesh sieve, mixed with 90 mL of distilled water in a 1:30 ratio, and placed in a water bath with boiling water for 2 h. The sample was cooled to room temperature before being centrifuged (6000 r/min) for 10 min to remove the precipitation and collect the supernatant. The pH was adjusted to 7, then 3% trichloroacetic acid solution (7.5% of the sample volume) was added, the sample was shaken well, and the absorbance was measured at 490 nm to calculate the polysaccharide content using the glucose standard curve.

### 2.11. The Presence of Allantoin

UV-visible high-performance liquid chromatography was used to determine allantoin content [26]. A total of 2 g of yam sample was weighed accurately, crushed, ground into powder, passed through a 60-mesh screen, and dissolved in 10 mL of distilled water. Then, 10~15 times of 95% ethanol was added, and the sample was sonicated at 4 °C for 10 min, before 10 mL of sample was taken, centrifuged (7000 r/min) for 10 min. Allantoin was evaluated using liquid chromatography. HPLC column: 5 μm, 250 mm × 4.6 mm; flow rate: 0.2 mL/min; UV wavelength: 200 nm; mobile phase: EtOH/CHCL_3_/H_2_O (0.5/0.012/100) were the conditions used for the colorimetric measurement of allantoin concentration.

### 2.12. Comprehensive Analysis Using the Coefficient of Variation Method

The coefficient of variation weighting method [27] was used in this work to measure the color difference values, rehydration rate, and polysaccharide and allantoin contents of yam slices dried under various circumstances.

Equation (10) is used to compute the coefficient of variation.

(10) $$V_{i}=\frac{\sigma_{i}}{x_{i}}$$

Equation (11) shows the formula for computing the weight of each sample index.

(11) $$W_{i}=\frac{V_{i}}{\sum_{i=1}^{n}V_{i}}$$

where *V_i_* is the coefficient of variation of the *i*th indicator, *W_i_* is the weight of the *i*th indicator, *σ_i_* is the standard deviation of the *i*th indicator, and *x_i_* is the mean of the *i*th indicator.

Following that, the data for each index were standardized as shown in Equation (12).

(12) $$Z_{i}=\frac{x_{j}-x_{i}}{\sigma_{i}}$$

where *Z_i_* is the value of each index after standardization; *x_j_* is the experimental measurement value of each index.

The quality of the color difference value is negatively connected, which means that the smaller the value, the better, so the negative sign must be inserted after standardization. Finally, the overall score is derived by multiplying the weights and normalized values of each indicator.

### 2.13. Model of Thin Layer Drying

Six thin-layer drying models were chosen to describe the drying curves at different temperatures. Table 1 shows the most popular thin-layer drying models.

The three parameters of correlation coefficient *R*^2^, chi-square *χ*^2^, and root mean square error *RMSE* [33] were used to compare the model’s applicability to the data. The *R*^2^ represents the similarity of the individual variables, and the closer the value is to 1, the more applicable the model is. The *χ*^2^ reaction to the deviation of the two variables, predicted and tested, is used to assess model fit consistency; the smaller its value, the better the fit. The root mean square error (*RMSE*) is used to characterize the precise discrepancy between model predictions and actual observations. The higher the goodness of fit, the lower the value of *RMSE*. Equations (13)–(15) are used to calculate the three statistical indicators mentioned above.

(13) $$R^{2}=1-\frac{\sum_{i=1}^{N}{\left(MR_{pre,i}-MR_{exp,i}\right)}^{2}}{\sum_{i=1}^{N}{\left(MR_{pre,a}-MR_{exp,i}\right)}^{2}}$$

(14) $$RMSE={\left[\frac{1}{N}\sum_{i=1}^{N}{\left({MR}_{pre,i}-{MR}_{exp,i}\right)}^{2}\right]}^{\frac{1}{2}}$$

(15) $$\chi^{2}=\frac{\sum_{i=1}^{N}{\left({MR}_{exp,i}-{MR}_{pre,i}\right)}^{2}}{N-n}$$

where *MR_exp,i_* is the *i*th moisture ratio measured by the drying test; *MR_pre,i_* is the *i*th moisture ratio of the prediction model; and *M* and *n* are the number of data sets measured by the test and the number of constants in the model, respectively.

### 2.14. Statistical Analysis

Data are presented as the mean and standard deviation of three repeated measurements. An optimal experimental design based on a one-way test was used [34]. Data were analyzed using SPSS statistical software (version 21.0). Differences were considered statistically significant at *p* < 0.05.

## 3. Results and Analysis

### 3.1. Drying Characteristics of Yam Slices

**Figure 4:**
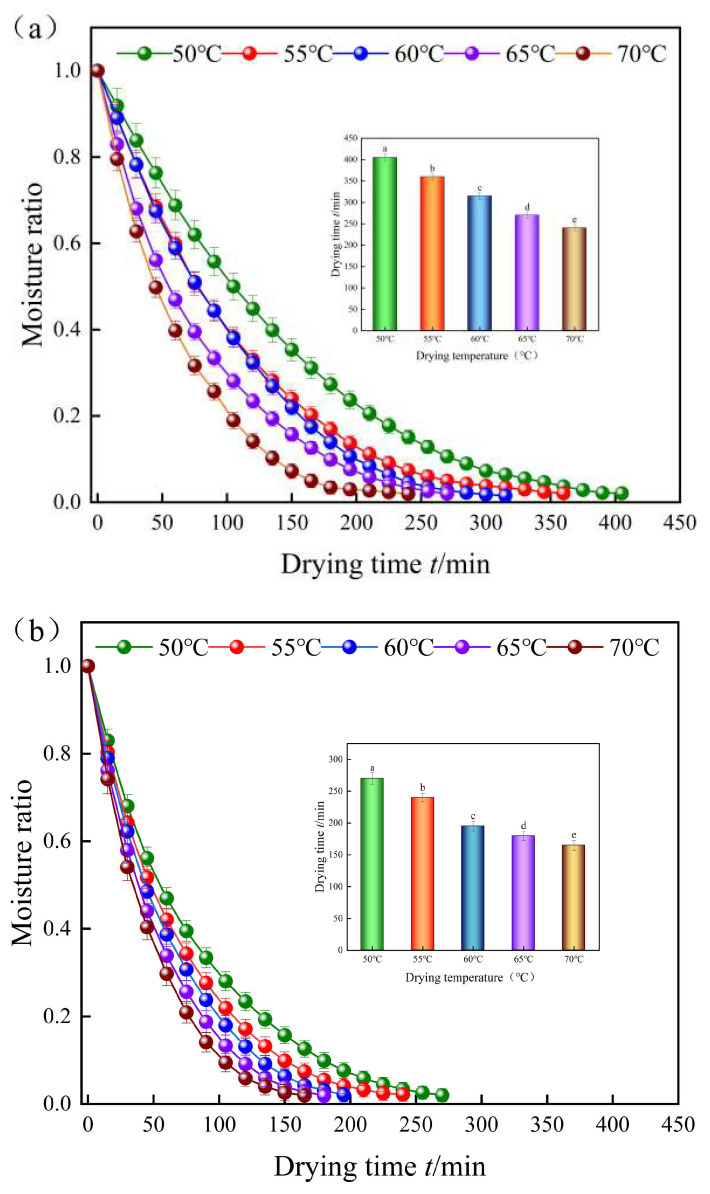
Moisture ratio curves of yam slices at different temperatures. HAD (**a**), IR-HAD (**b**). Note: Different letters in the graphs show significant differences according to the Duncan test (*p* < 0.05).

Figure 4 depicts the drying time and moisture ratio (MR) of yam slices for hot air drying (a) and combined infrared hot air drying (b) at various temperatures. The figure shows that varied drying temperatures had a substantial impact on the MR variation of yam slices in all cases. The moisture ratio in the yam slices decreases as the drying time increases and falls faster as the temperature rises. The time necessary for IR-HAD of yam slices to the desired moisture content was 270, 240, 195, 180, and 165 min at the same drying temperature (50 °C, 55 °C, 60 °C, 65 °C, and 70 °C), while the time required for HAD was 405, 360, 315, 270, and 240 min. The time required for IR-HAD was 31.25~38.1% less than that required for HAD. This research demonstrates that boosting the radiant heat transmission to HAD enhances moisture evaporation and diffusion, resulting in a reduced drying time.

**Figure 5:**
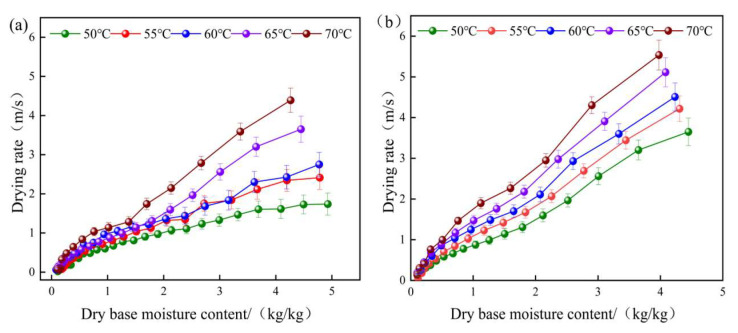
Drying rate vs. moisture content curves of yam slices at different temperatures. HAD (**a**), IR-HAD (**b**).

The drying rate vs. moisture content curves of yam slices at different temperatures are shown in Figure 5 for hot air drying (a) and combined infrared hot air drying (b). The drying temperature has a substantial effect on the drying rate (DR) of yam slices in IR-HAD and HAD, as shown in the figure, and a greater drying temperature can increase the DR. The drying rate is fast in the beginning and slows down in the later stages. The entire stage is characterized by a decreasing drying rate, with no discernible phase of steady drying rate. This is primarily because yam slices become progressively less watery during drying and cannot deliver a steady supply of water, and diffusion is the primary physical mechanism directing the flow of water from the interior to the surface of yam slices during IR-HAD and HAD [35].

At drying temperatures of 50 °C, 55 °C, 60 °C, 65 °C, and 70 °C, the DRs of IR-HAD and HAD were 0.0526, 0.122, 0.1396, 0.1906, and 0.2063 g/g·min and 0.0193, 0.0643, 0.0686, 0.0793, and 0.1322 g/g·min, respectively. The DR of IR-HAD is more than 1.56 times that of HAD at the same temperature. This is due to HAD’s inability to rapidly elevate the interior temperature of the yam slices. Furthermore, IR radiation in IR-HAD may penetrate the surface layer of yam slices and generate additional heat within them, allowing the desired temperature to be reached rapidly and the drying rate to rise. By analyzing turmeric tablets, Jeevarathinam et al. [36] came to the same conclusion.

### 3.2. Effective Moisture Diffusion Coefficient

The linear equations and values of the effective moisture diffusion coefficients of IR-HAD and HAD for yam slices at different temperatures are shown in Table 2 and Table 3, respectively. The effective water diffusion coefficients of both IR-HAD and HAD showed a rising trend, as shown in the table. The *Deff* at 70 °C is more than 1.8 times that of 50 °C in both drying procedures. This is because the greater temperature accelerates the evaporation of water molecules in yam slices, which in turn promotes the diffusion of water in yam slices [37]. Furthermore, at the same temperature, the *Deff* of IR-HAD was higher than that of HAD. This could be because the greater *Deff* is mostly due to the fast heating of IR radiation during IR-HAD [38].

### 3.3. Yam Slice Activation Energy

**Figure 6:**
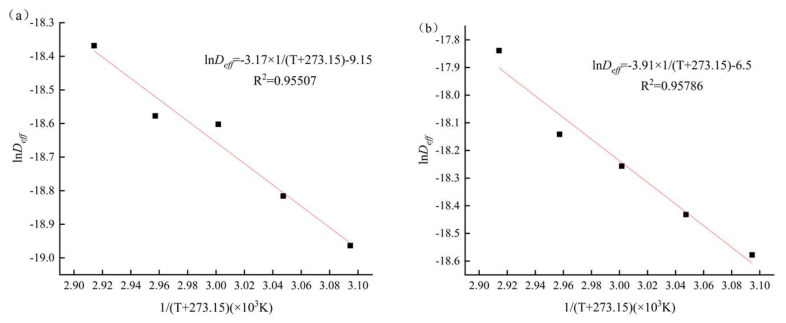
Effective moisture diffusion coefficient of yam slices vs. drying temperature. IR-HAD (**a**), HAD (**b**). Note: The red line indicates the fitted curve and the equation is the fitted equation.

The drying activation energy is an important metric for determining the difficulty of the drying process. The lower the value, the simpler it is to dry. Figure 6 depicts the connection between ln(*D_eff_*) and 1/(T + 273.15) in IR-HAD and HAD drying. According to the Arrhenius formula (Equation (6)), the slope of the linear equation produced from the fit was (−E_a_/R), the intercept was ln(D_0_), and the activation energies of IR-HAD and HAD of yam slices were determined as 26.35 kJ/mol and 32.53 kJ/mol, respectively. IR-HAD utilizes infrared radiation to act directly on the moisture molecules inside the yam chips to accelerate the evaporation process and reduce energy consumption. Compared with HAD, IRHAD utilizes the high thermal effect of infrared radiation to rapidly heat the moisture molecules, shortening the drying time and reducing the activation energy. IRHAD uses infrared radiation heating and hot air circulation to make the drying time shorter and reduce the reaction time. According to the Arrhenius formula, when the reaction time is reduced, the reaction rate constant increases, and the activation energy is smaller. This shows that adding infrared radiation to the HAD may make drying easier.

### 3.4. Unit Energy Consumption

**Figure 7:**
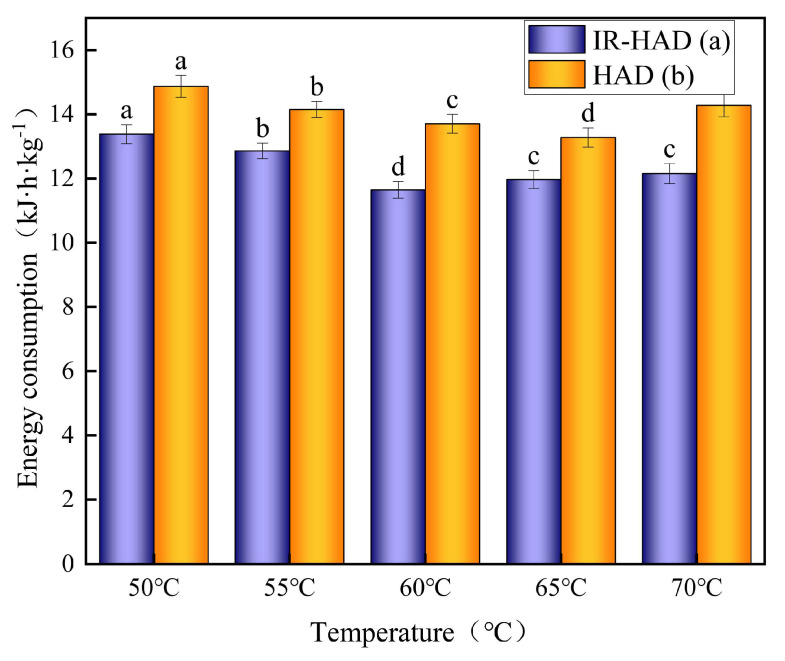
Unit energy consumption of yam slices at different temperatures. IR-HAD (a), HAD (b). Note: Different letters in the graphs show significant differences according to the Duncan test (*p* < 0.05).

The unit energy consumption is a measure of the energy consumed by the equipment and enables the evaluation of the equipment’s energy efficiency and potential for energy savings [39]. The unit energy consumption of IR-HAD (a) and HAD (b) for yam slices at different temperatures is shown in Figure 7. As shown in the graph, the unit energy consumption of both drying processes increases and subsequently reduces as the temperature rises. Because hot air at increased temperatures has a better heat transfer efficiency, the heat transfer rate to yam slices is also faster, implying that the same drying effect can be obtained in a shorter period of time, resulting in decreased energy consumption per unit. A temperature of 70 °C, on the other hand, can induce a hard coating to form on the surface of the yam slices, limiting water evaporation. Furthermore, increasing the temperature raises the dryer’s power requirement, resulting in an increase in energy consumption per unit. At the same temperature, the energy consumption per unit of HAD exceeds that of IR-HAD by more than 1.3 times. This is most likely due to the fact that IR-HAD uses infrared radiation to convey heat directly to the yam slices, causing them to absorb heat and evaporate water more quickly. And hot air drying requires convective heat transfer, which reduces drying efficiency and energy consumption.

### 3.5. Color Evaluation

**Figure 8:**
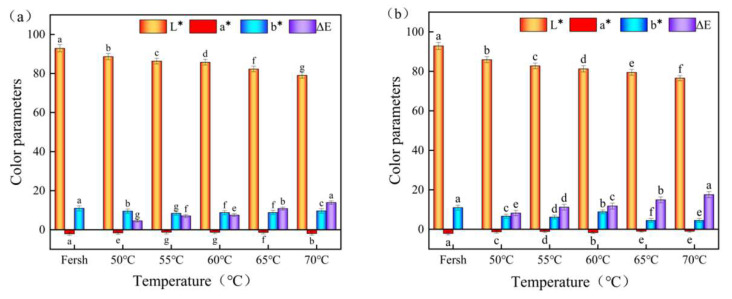
Color of yam slices at different temperatures. IR-HAD (**a**), HAD (**b**). Note: Different letters in the graphs show significant differences according to the Duncan test (*p* < 0.05).

The color of yam slices is a crucial indicator of drying quality, which influences consumer choice and value assessment [40]. Figure 8 depicts the IR-HAD (a) and HAD (b) color indices for yam slices at various temperatures. When compared to fresh yam, the color characteristics of yam slices treated with both drying procedures were dramatically reduced. At the same temperature, the brightness (L) of IR-HAD is greater than that of HAD and diminishes with the increase in temperature. When compared to IR-HAD, HAD dramatically reduced the yellow-blue values (b) of yam slices. The red and green values (a) did not differ significantly between the two drying procedures. At drying temperatures of 50 °C, 55 °C, 60 °C, 65 °C, and 70 °C, the color difference values of IR-HAD were 4.52, 7.08, 7.49, 10.85, and 13.91, while the color difference values of HAD were 8.24, 11.2, 11.79, 14.92, and 17.57, respectively. At the same temperature, the color difference values of HAD were all greater than those of IR-HAD, most likely because IR-HAD uniformly distributes the surface and internal temperature distribution of yam slices, eliminating the local overheating issue produced by uneven heat transfer in HAD. Furthermore, IR-HAD has a shorter drying time than HAD, limiting the exposure of yam slices to high temperatures and thereby lowering pigment oxidation.

The color contrast between yam slices grew progressively as the temperature rose. The Δ*E* at 70 °C was more than 2.1 times higher than that at 50 °C in both drying methods. When the temperature rises, the movement within the molecules is intense, which leads to an increase in the destruction of pigments, enzymes, and other substances in the yam slices, causing the color of the slices to increase as a result. Zhang et al. [41] found that lower temperatures were able to reduce the color difference of dry products to some extent. At lower temperatures, the chemical reaction rate of the dried products was slowed down, thus reducing the possibility of color change. In addition, lower temperatures also help retain the natural pigments and nutrients in dried products, further reducing the occurrence of color shifts.

### 3.6. Rehydration Rate

**Figure 9:**
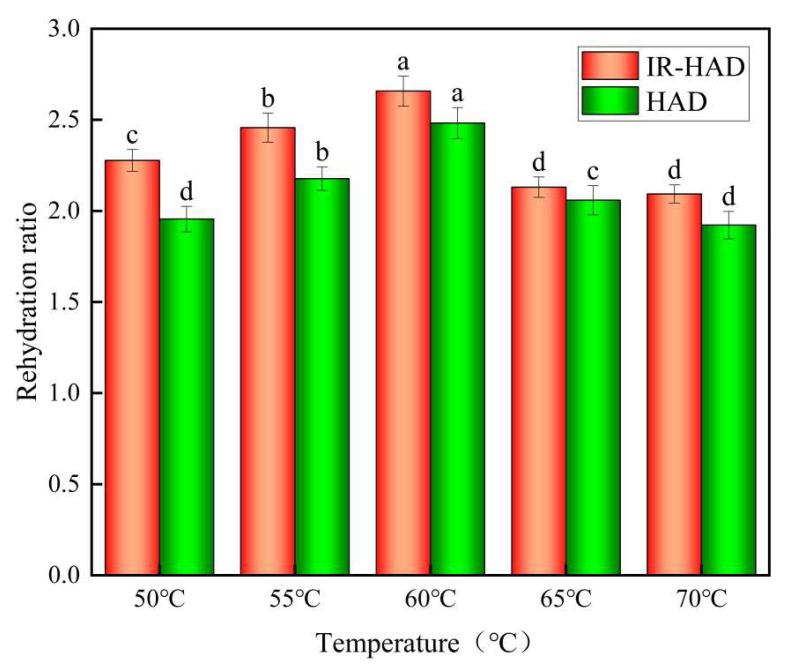
Rehydration ratios of thin slices of yam at different temperatures. Note: Different letters in the graphs show significant differences according to the Duncan test (*p* < 0.05).

A faster rehydration rate means less internal damage to the product, reflecting a higher level of product quality [42]. Figure 9 depicts the rate of rehydration of IR-HAD and HAD at various temperatures of yam slices. The figure shows that the rehydration rate of both IR-HAD and HAD increased first and then dropped as the drying temperature increased. At 60 °C, the greatest rehydration rate was 2.66 and 2.48, respectively. When the drying temperature increases, yam slices absorb more energy per unit mass, leading to an increase in vapor pressure inside the tissue, which in turn leads to an increase in tissue swelling and rehydration rate of yam slices. When the drying temperature exceeds a particular threshold, the drying rate of yam slices is accelerated, causing the surface structure to gradually harden and, as a result, the rehydration rate to decrease [43]. At the same temperature, the rehydration rates of IR-HAD were all higher than those of HAD. This implies that infrared radiation can significantly improve the water absorption characteristics of yam slices, allowing for rapid water content recovery during rehydration.

### 3.7. Microstructure

**Figure 10:**
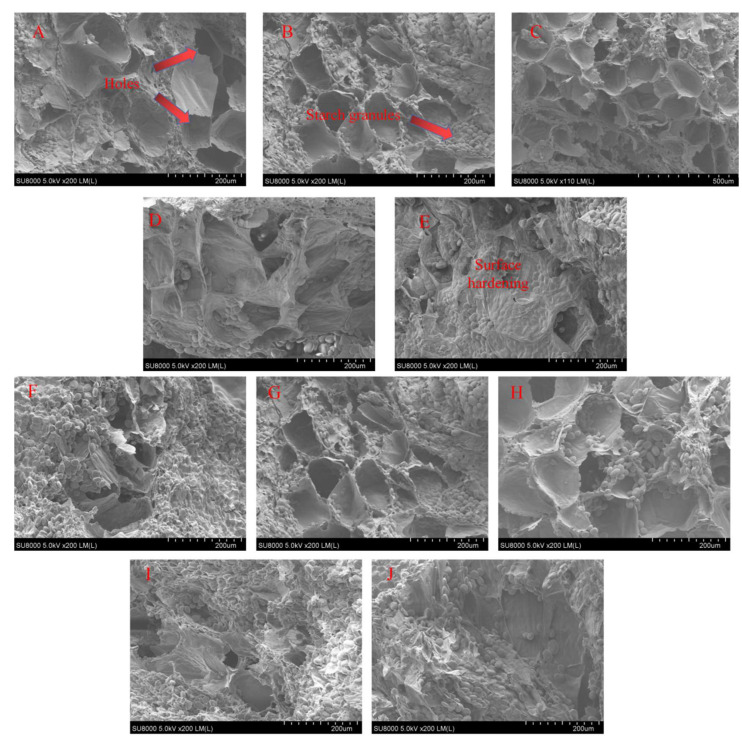
Microstructure of yam slices at different temperatures. IR-HAD: 50 °C (**A**), 55 °C (**B**), 60 °C (**C**), 65 °C (**D**), 70 °C (**E**); HAD: 50 °C (**F**), 55 °C (**G**), 60 °C (**H**), 65 °C (**I**), 70 °C (**J**).

The microstructure of yam slices at different temperatures of IR-HID and HAD is shown in Figure 10. The presence of holes of different sizes and shapes on the cut surface of yam slices resulted in the outflow of some starch granules, which is similar to the microstructure of potato slices [44]. The porosity increased with the increase in drying temperature, initially increasing and then decreasing. This is because raising the temperature causes the interior tissue of yam slices to swell and facilitates the production of more pores, but raising the temperature too high causes the slices to harden and the pores to collapse, lowering porosity. Furthermore, IR-HID has a more homogeneous microstructure than HAD. This is due to the infrared radiation and hot air heat both on the inside and exterior of the yam slices, making water evaporation more uniform and favorable to porosity development [45].

### 3.8. Polysaccharide Content

**Figure 11:**
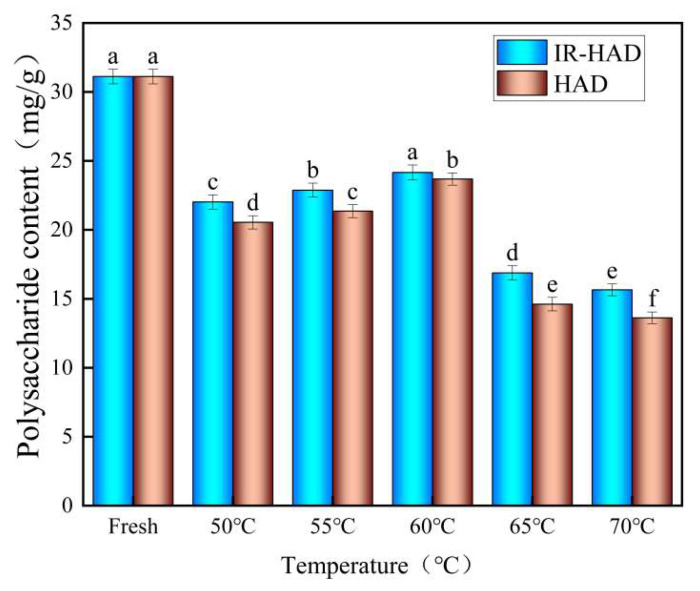
Polysaccharide content of yam slices at different temperatures. Note: Different letters in the graphs show significant differences according to the Duncan test (*p* < 0.05).

Polysaccharides are thermosensitive molecules that contain hypoglycemic, antioxidant, anticancer, and immunological boosting properties, making them key active components of yam [25]. Figure 11 depicts the effects of IR-HID and HAD on yam polysaccharides at various temperatures. The figure shows that drying temperature has a substantial impact on yam polysaccharides. Fresh yam has a polysaccharide content of 36.72 mg/g. After drying at 50 °C, 55 °C, 60 °C, 65 °C, and 70 °C, the polysaccharide content of IR-HID reduced by 29.23%, 26.48%, 23.35%, 45.76%, and 49.7%, respectively, and the polysaccharide content of HAD decreased by 33.97%, 31.38%, 23.89%, 53.04%, and 56.24%. The maximum polysaccharide content was found at 60 °C for both drying techniques, with values of 24.17 mg/g and 23.68 mg/g, respectively. This is due to the fact that increasing the temperature within a given temperature range aids in the breakdown of chemical bonds between yam polysaccharide molecules, resulting in novel reactive groups, conformations, and chemical connections. This increases the polysaccharide composition and thus the polysaccharide content. However, polysaccharides are temperature sensitive, and high temperatures can cause polysaccharide breakdown, resulting in a drop in polysaccharide concentration. Zheng and colleagues [41] discovered that the polysaccharide content of winter wheat grew and then dropped when the drying temperature was between 50 and 70 °C, with the maximum level found at 60 °C, validating the polysaccharide features. The polysaccharide content of IR-HID is generally higher than that of HAD, which may be due to the microwave effect of infrared radiation, in which the water molecules inside the yam slices rub and vibrate, generating heat and promoting water evaporation while reducing polysaccharide oxidation with air.

### 3.9. The Presence of Allantoin

**Figure 12:**
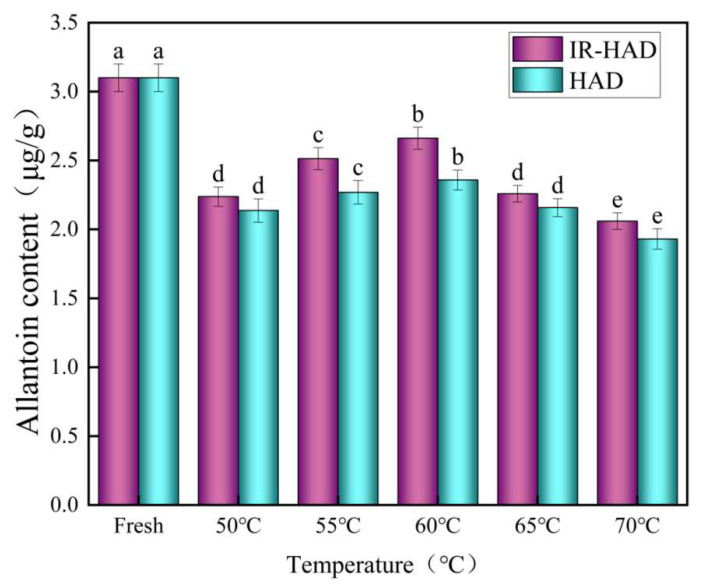
Allantoin content of yam slices at different temperatures. Note: Different letters in the graphs show significant differences according to the Duncan test (*p* < 0.05).

Allantoin contains anti-inflammatory, antioxidant, keratolytic, and skin softening properties [46]. Figure 12 depicts the effect of IR-HID and HAD on yam chip allantoin at various drying temperatures. The allantoin content in dried treated yam slices ranged from 1.93 to 2.66 g/g, as shown in the figure. The allantoin content increased with the increase in drying temperature, initially increasing and then decreasing. This is due to the fact that excessive drying time can result in allantoin loss. At the same time, because allantoin is easily soluble in water, if the drying temperature is too high, the quick loss of water may result in the loss of allantoin, leading the content to plummet. In general, the allantoin content of IR-HID was higher than that of HAD. IR-HID yam slices use infrared radiation technology to heat and progressively harden the yam slice’s interior. Water evaporation lowers allantoin loss during this procedure.

### 3.10. Comprehensive Analysis Using the Coefficient of Variation Method

The coefficient of variation technique may objectively describe the overall quality of yam slices, and Table 4 shows that the color difference value accounted for the most weight (0.48), while the allantoin content accounted for the least weight (0.12).

**Figure 13:**
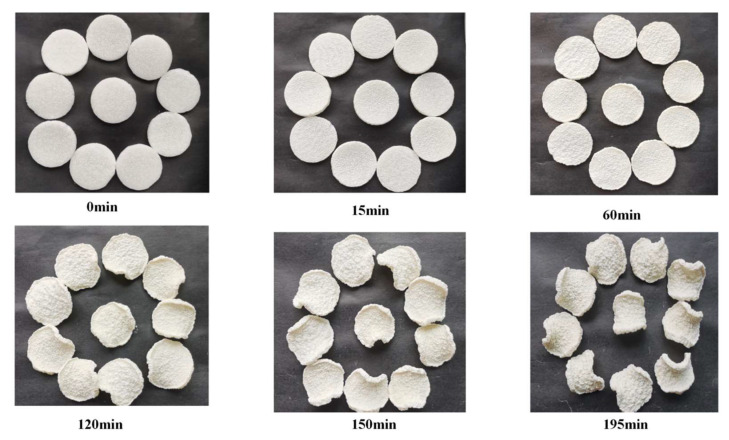
Experimental process of IR-HAD yam slices at drying temperature of 60 °C.

According to the total quality score in Table 5, the IR-HAD at 60 °C has the best quality, with a value of 1.26. At 70 °C, the poorest HAD quality was −1.63. Figure 13 shows the drying test of yam slices when the overall quality is optimal.

### 3.11. Drying Kinetic Curve of Yam Slices

Six thin-layer drying models were fitted with moisture ratios (MRs). The coefficient of determination (*R*^2^), chi-square (*χ*^2^), and root mean square error (*RMSE*) were used to compare the quality of fit of the evaluated IR-HAD and HAD. Table 6 and Table 7 illustrate the parameter values and statistical findings of the drying models.

Table 6 shows that at 50, 55, 60, 65, and 70 °C, the *R*^2^, *RMSE*, and *χ*^2^ values ranged from 0.99757 to 0.99936, 7.06 × 10^−4^ to 0.003, and 5.64 × 10^−5^ to 9.91 × 10^−4^, respectively. At 50, 55, 60, 65, and 70 °C, compared with the Lewis model, the Page model, Henderson and Pabis model, Verma model, and two-term exponential, the Weibull model had the highest R^2^ (0.99887~0.99936) and the lowest RMSE values (8.696 × 10^−4^~0.0163) and *χ*^2^ values (5.64 × 10^−5^~1.71 × 10^−4^). This was followed by the *R*^2^ (0.99866~0.99927), *RMSE* (8.696 × 10^−4^~0.00104), and *χ*^2^ (9.91 × 10^−5^~6.91 × 10^−4^) of the Page model. Therefore, the Weibull model best expresses the changes in the moisture ratio of mountain tablets during infrared radiation at different infrared hot air temperatures (50~70 °C).

Table 7 shows that the range of *R*^2^, *RMSE*, and *χ*^2^ values for all models during HAD was 0.98816~0.99963, 7.56 × 10^−4^~0.02614, and 3.27 × 10^−5^~1.3 × 10^−4^, respectively. at 50, 55, 60, 65, and 70 °C, compared to the Lewis model, Page model, Henderson and Pabis model, Verma model, and two-term exponential, the Weibull model had the highest *R*^2^ (0.99812~0.99963) and the lowest *RMSE* values (7.56 × 10^−4^~0.00247) and χ^2^ values (3.71 × 10^−5^~1.88 × 10^−4^). This was followed by the Page model with *R*^2^ (0.99802~0.99958), *RMSE* (8.56 × 10^−4^~0.00377), and *χ*^2^ (3.72 × 10^−5^~1.89 × 10^−4^). Therefore, the Weibull model best expresses the changes in the moisture ratio of mountain tablets during infrared radiation at different infrared hot air temperatures (50~70 °C).

**Figure 14:**
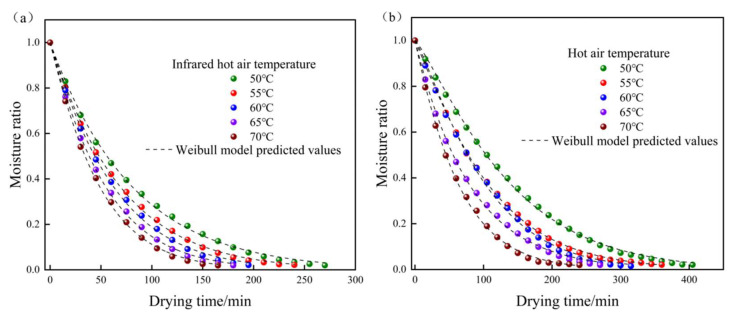
Comparison of predicted and experimental values of Weibull model at different temperatures. IR-HAD (**a**), HAD (**b**).

Figure 14 depicts the experimental moisture ratio values and predicted Weibull model values for yam slices dried at various temperatures (50 °C, 55 °C, 60 °C, 65 °C, and 70 °C). The experimental and projected values fit well in both drying processes, and the Weibull model well describes the changing of moisture ratio of yam slices under various conditions.

## 4. Conclusions

Hot air drying (HAD) and combined infrared hot air drying (IR-HAD) experiments on yam slices at 50 °C, 55 °C, 60 °C, 65 °C, and 70 °C were performed in this study, and the indices of drying kinetics, unit energy consumption, and nutrient composition were examined. The outcomes were as follows:

(1) Yam slices are progressively dried throughout the IR-HAD and HAD stages; there is no fixed constant temperature drying rate period, and temperature increases can encourage moisture transfer. At the same drying temperature, IR-HAD needed 31.25~38.1% less time than HAD, and the drying rate of IR-HAD was more than 1.56 times that of HAD.
(2) The *D_eff_* of IR-HAD is higher than that of HAD at the same temperature, and it increases with temperature, with the *D_eff_* at 70 °C being more than 1.8 times that of 50 °C. IR-HAD has a lower activation energy of 26.35 kJ/mol than HAD, which has a higher activation energy of 32.53 kJ/mol.
(3) The unit energy consumption of both drying processes increased initially and subsequently dropped as the temperature climbed. Furthermore, at the same temperature, HAD has a larger unit energy consumption than IR-HAD, more than 1.3 times higher.
(4) As the drying temperature climbed, the color difference value grew, and the ΔE at 70 °C was more than 2.1 times that at 50 °C. The rehydration rate, microscopic porosity, and polysaccharide and allantoin content all increased and then decreased with the increase in temperature. IR-HAD-treated yam slices outperformed HAD in all five quality metrics at the same temperature. At 60 °C, IR-HAD produced the finest overall quality of yam slices.
(5) Six thin-layer drying models describing yam slices were fitted and compared with the test value data, and three goodness-of-fit assessment indices revealed that the Weibull model was more compatible with the variation pattern of the drying test data.

## Figures and Tables

**Table 1:** Mathematical model for the fitting of the drying curve.

Serial Number	Model Name	Model Equations	References
1	Lewis model	*MR* = exp(−kt)	[28]
2	Page model	*MR* = exp(−kt)*^n^*	[29]
3	Henderson and Pabis model	*MR* = *a*exp(−kt)	[30]
4	Verma model	*MR* = *a*exp(−kt) + (1 − *a*)exp(−gt)	[31]
5	Two-term model	*MR* = *a*exp(−kt) + (1 − *a*)exp(−*ka*t)	[5]
6	Weibull model	*MR* = exp(−(*t/α*)*^β^*)	[32]

**Table 2:** Linear regression equations and effective moisture diffusion coefficients of IR-HAD at different temperatures.

Temperature (°C)	Linear Equation	*R* ^2^	*D_eff_* (m^2^/s)
50	LnMR = −0.2375t + 0.1338	0.99051	8.55 × 10^−9^
55	LnMR = −0.2747t + 0.1072	0.99605	9.89 × 10^−8^
60	LnMR = −0.3275t + 0.175	0.98857	1.18 × 10^−8^
65	LnMR = −0.3673t + 0.1585	0.99189	1.32 × 10^−8^
70	LnMR = −0.4097t + 0.1467	0.99437	1.79 × 10^−8^

**Table 3:** Linear regression equation and effective moisture diffusion coefficient of HAD at different temperatures.

Temperature (°C)	Linear Equation	*R* ^2^	*D_eff_* (m^2^/s)
50	LnMR = −0.1615t + 0.2792	0.98352	5.81 × 10^−9^
55	LnMR = −0.1871t + 0.1491	0.99622	6.74 × 10^−9^
60	LnMR = −0.2317t + 0.33	0.9825	8.34 × 10^−9^
65	LnMR = −0.2375t + 0.1338	0.99051	8.55 × 10^−9^
70	LnMR = −0.2927t + 0.0772	0.99024	1.05 × 10^−8^

**Table 4:** Weight of each indicator.

Indicators	Standard Deviation	Standard Deviation	Coefficient of Variation	Weights
Color difference	3.8	10.76	0.35	0.48
Rehydration rate	0.23	2.22	0.1	0.14
Polysaccharide content	3.76	19.54	0.19	0.26
Allantoin content	0.2	2.26	0.09	0.12

**Table 5:** Comprehensive evaluation of the quality of yam slices.

Indicators	IR-HAD 50 °C	IR-HAD 55 °C	IR-HAD 60 °C	IR-HAD 65 °C	IR-HAD 70 °C	HAD 50 °C	HAD 55 °C	HAD 60 °C	HAD 65 °C	HAD 70 °C
Color difference	0.79	0.46	0.41	−0.01	−0.4	0.32	−0.06	−1.3	−0.53	−0.86
Rehydration rate	0.03	0.14	0.27	−0.05	−0.08	−0.16	−0.03	0.16	−0.1	−0.18
Polysaccharide	0.17	0.23	0.32	−0.18	−0.27	0.07	0.13	0.29	−0.34	−0.41
Allantoin content	0.01	0.17	0.26	0.02	−0.1	−0.06	0.02	0.08	−0.04	−0.18
Overall rating	1	1.01	1.26	−0.23	−0.85	0.17	0.07	0.39	−1.01	−1.63

**Table 6:** Parameter values and statistical results of drying models at different infrared hot air temperatures.

Models	Temperature (°C)	Model Constants	*R* ^2^	*RMSE*	*χ* ^2^
Lewis model	50	k = 0.01254	0.99879	0.00185	9.56 × 10^−4^
	55	k = 0.01254	0.99893	0.00151	9.44 × 10^−4^
	60	k = 0.0164	0.99757	0.003	2.31 × 10^−4^
	65	k = 0.0187	0.99815	0.00216	1.8 × 10^−4^
	70	k = 0.021	0.99797	0.00225	2.05 × 10^−4^
Page model	50	k = 0.01183, *n* = 1.01271	0.99884	0.00177	1.04 × 10^−4^
	55	k = 0.01268, *n* = 1.03428	0.99927	0.00104	6.91 × 10^−4^
	60	k = 0.01229, *n* = 1.06699	0.99866	0.00153	1.27 × 10^−4^
	65	k = 0.01454, *n* = 1.06	0.99898	0.00109	9.91 × 10^−5^
	70	k = 0.01559, *n* = 1.07	0.99915	8.56 × 10^−4^	8.56 × 10^−5^
Henderson and Pabis model	50	k = 0.01255, a = 1.00035	0.99879	0.00185	1.09 × 10^−4^
	55	k = 0.01484, a = 1.00559	0.9989	0.00146	9.71 × 10^−4^
	60	k = 0.0166, a = 1.0012	0.99776	0.00276	2.3 × 10^−4^
	65	k = 0.01899, a = 1.01	0.99812	0.00201	1.83 × 10^−4^
	70	k = 0.02126, a = 1.012	0.99798	0.00204	2.04 × 10^−4^
Verma model	50	k = 0.0125, a = 1, b = 11.86	0.99864	0.00185	1.16 × 10^−4^
	55	k = 0.01495, a = 1.01, b = 10.87	0.99889	0.00138	9.86 × 10^−5^
	60	k = 0.01693, a = 1.03, b = 10.39	0.99774	0.00236	2.15 × 10^−4^
	65	k = 0.01927, a = 1.03, b = 9.48	0.99824	0.00171	8.14 × 10^−4^
	70	k = 0.02189, a = 1.04, b = 8.88	0.99834	0.00151	1.68 × 10^−4^
Two-term exponential model	50	k = 0.0136, a = 1.3218	0.99884	0.00177	1.04 × 10^−4^
	55	k = 0.01666, a = 1.408	0.99922	0.00104	6.91 × 10^−5^
	60	k = 0.01944, a = 1.502	0.99866	0.00153	1.27 × 10^−4^
	65	k = 0.02196, a = 1.48	0.99898	0.00109	9.91 × 10^−4^
	70	k = 0.02506, a = 1.51	0.99915	8.56 × 10^−4^	8.56 × 10^−5^
Weibull model	50	α = 79.94, β = 1.01319	0.99887	0.00163	1.03 × 10^−4^
	55	α = 68.26, β = 1.0348	0.99936	8.416 × 10^−4^	5.64 × 10^−5^
	60	α = 61.74, β = 1.067	0.99887	0.00129	1.08 × 10^−4^
	65	α = 54.09, β = 1.0607	0.99919	8.696 × 10^−4^	1.71 × 10^−4^
	70	α = 48.29, β = 1.0737	0.9993	7.06 × 10^−4^	1.68 × 10^−4^

**Table 7:** Parameter values and statistical results of the drying model at different hot air temperatures.

Models	Temperature (°C)	Model Constants	*R* ^2^	*RMSE*	*χ* ^2^
Lewis model	50	k = 0.00782	0.98931	0.02614	9.68 × 10^−4^
	55	k = 0.00953	0.99495	0.01071	4.46 × 10^−4^
	60	k = 0.00998	0.98816	0.02367	0.00113
	65	k = 0.01254	0.99879	0.00185	1.04 × 10^−4^
	70	k = 0.01589	0.99802	0.00286	1.78 × 10^−4^
Page model	50	k = 0.00272, *n* = 1.1954	0.99895	0.00247	9.51 × 10^−5^
	55	k = 0.00497, *n* = 1.1343	0.99958	8.56 × 10^−4^	3.72 × 10^−5^
	60	k = 0.00377, *n* = 1.2	0.99802	0.00377	1.89 × 10^−4^
	65	k = 0.01183, *n* = 1.013	0.99877	0.00177	1.05 × 10^−4^
	70	k = 0.01266, *n* = 1.06	0.99885	0.00156	1.04 × 10^−4^
Henderson and Pabis model	50	k = 0.00766, a = 1.053	0.99231	0.0181	9.96 × 10^−4^
	55	k = 0.009896, a = 1.039	0.99645	0.00721	3.13 × 10^−4^
	60	k = 0.01047, a = 1.05	0.9906	0.0179	8.95 × 10^−4^
	65	k = 0.01255, a = 1	0.99872	0.0185	1.09 × 10^−4^
	70	k = 0.01606, a = 1.01	0.99805	0.00264	1.76 × 10^−4^
Verma model	50	k = 0.00789, a = 1.08, b = 15.94	0.994	0.01359	5.43 × 10^−4^
	55	k = 0.01018, a = 1.07, b = 14.58	0.99769	0.00448	2.03 × 10^−4^
	60	k = 0.01089, a = 1.094, b = 14.5	0.99343	0.01314	6.91 × 10^−4^
	65	k = 0.01255, a = 1, b = 11.86	0.99864	0.00185	1.16 × 10^−4^
	70	k = 0.01635, a = 1.03, b = 10.59	0.99817	0.0023	1.65 × 10^−4^
Two-term exponential model	50	k = 0.00979, a = 1.728	0.99886	0.00268	1.03 × 10^−4^
	55	k = 0.01221, a = 1.64	0.99958	8.55 × 10^−4^	3.29 × 10^−5^
	60	k = 0.013431, a = 1.74	0.99789	0.00423	2.11 × 10^−4^
	65	k = 0.0136, a = 1.32	0.99877	0.00177	9.56 × 10^−4^
	70	k = 0.0186, a = 1.48	0.99885	0.00156	8.84 × 10^−4^
Weibull model	50	α = 141.19, β = 1.19	0.99895	0.00247	9.5 × 10^−5^
	55	α = 107.4, β = 1.14	0.99963	7.56 × 10^−4^	3.71 × 10^−5^
	60	α = 103.5, β = 1.21	0.99812	0.00376	1.88 × 10^−4^
	65	α = 79.94, β = 1.01	0.99887	0.00163	1.03 × 10^−4^
	70	α = 63.73, β = 1.06	0.99908	0.00133	1.04 × 10^−4^

## Data Availability

The datasets generated for this study are available upon request from the corresponding author.
